# Outcomes of young people who reach the transition boundary of child and adolescent mental health services: a systematic review

**DOI:** 10.1007/s00787-019-01307-7

**Published:** 2019-03-08

**Authors:** Rebecca Appleton, Catriona Connell, Emma Fairclough, Helena Tuomainen, Swaran P. Singh

**Affiliations:** 1 Warwick Medical School, University of Warwick, Coventry, CV4 7AL UK; 2 Institute of Psychological Sciences, University of Leeds, Leeds, LS2 9JT UK

**Keywords:** Transition, CAMHS, AMHS, Mental health service, Systematic review

## Abstract

When young people reach the upper age limit of child and adolescent mental health services (CAMHS), care should be transferred to an adult mental health service (AMHS) if they require ongoing support. However, many young people experience a significant disruption of their care during this transition, whilst others may fail to transition at all. Currently, there is no systematic appraisal of the international evidence regarding the outcomes of young people after transition. A systematic review was conducted which aimed to synthesise and review the existing research regarding outcomes after transition. We searched six databases from their inception until December 2017 for research relating to either the mental health or service use outcomes of young people after reaching their CAMHS age boundary. Results were synthesised narratively. The initial searches identified 18,287 papers, of which 213 were screened on full text. 13 papers were included in the review, representing 10 cohorts of young people who crossed the transition age boundary. No studies contained extractable data on mental health outcomes following transition, and therefore, this review focused only on service use outcomes. Results showed a quarter of young people transitioned to AMHS, with the other young people experiencing varied outcomes after leaving CAMHS and multiple transitions during this time. This review provides evidence for the varying service use outcomes of young people after reaching the upper age limit of CAMHS. However, longitudinal research into long-term outcomes is lacking, in addition to research regarding the mental health and functioning outcomes of young people following transition.

*Protocol registration* The protocol for this systematic review has been registered with PROSPERO, ID number CRD42018085916.

## Introduction

In high-income countries, mental health services are divided into separate specialties for children and adolescents, and adults. When young people reach the upper age limit of their child and adolescent mental health service (CAMHS), at around the age of 16–18 [1, 2], decisions need to be made regarding their future care. If a young person approaching this age boundary is judged to have an ongoing clinical need which requires specialist future treatment, care should be transferred to an adult mental health service (AMHS). This transfer of care should occur through part of the therapeutic process known as transition [3]. Four features of optimal transition have been identified: it should be planned well in advance and feature a joint meeting between both clinical teams and the young person; there should be a period of parallel care; all the young person’s information should be transferred to the new service and there should be continuity of care after the young person has left CAMHS [4]. However, the previous research has shown that these four features rarely occur, with one study estimating them to be present in only 4% of transitions [4].

Whilst some find the transition to AMHS difficult, some young people fail to transition at all, despite having an ongoing clinical need [5]. Some young people, although unwell, may not meet the eligibility thresholds for care at AMHS [6], which are often higher than those to access care at CAMHS. These differences in threshold are partly due to the differing approaches between the services [7], with CAMHS focusing on developmental and family problems, and AMHS specialising in the treatment of more severe chronic mental illnesses [8, 9]. The contrasting approaches between the services can also make it difficult for young people to adapt to care at AMHS [10], which could lead to poor engagement with their new service [11].

Young people who do not successfully transition to AMHS despite still needing care are said to have fallen through the gap between services. Currently, we do not know what happens to these young people, something which has been called a “serious cause for concern” [4] (p310) and highlighted as a significant gap in our knowledge in a recent report by the National Health Service (NHS) Healthcare Safety Investigation Branch [12] and in National Institute for Health and Care Excellence (NICE) transition guidelines [13].

To the best of our knowledge, no study has attempted to systematically review and robustly collate the evidence regarding the clinical and functioning outcomes (e.g., illness severity and living skills) of young people once they have reached the upper limit of their CAMHS service. The research questions for this review are as follows: (1) what are the service use destinations of young people after they reach the CAMHS age boundary? (2) what are the mental health outcomes of young people after they reach the CAMHS age boundary?

## Methods

This systematic review was conducted and reported in concordance with the PRISMA guidelines. The protocol for this review was registered with PROSPERO, ID number CRD42018085916.

### Search strategy

After the initial scoping searches, six bibliographic databases were searched (Medline, PsycINFO, CINAHL, Embase, and Web of Science) for the relevant literature from their inception until December 2017. Search terms were developed in collaboration with an information specialist, and contained terms relating to transition, young people, and mental health. An example search strategy can be found in Table 1. The reference lists of relevant systematic reviews which were identified during title and abstract screening were hand searched for additional relevant studies, although none were identified.

**Table 1:** An Example search strategy from Medline

#	Searches
1	continuity of care/ or exp transition to adult care/ or exp transitional care/ or care pathway.mp.
2	((transition or transfer* or continuity or interface) and care).mp.
3	1 or 2
4	mental health services.mp. or exp Mental Health Services/
5	mental health.mp. or exp Mental Health/
6	exp Mental Disorders/ or exp Psychiatry/ or psychiatr*.mp.
7	mental illness*.mp.
8	camhs.mp.
9	amhs.mp.
10	4 or 5 or 6 or 7 or 8 or 9
11	3 and 10
12	young adult.mp. or exp Young Adult/
13	exp Adolescent/ or adolescen*.mp. or exp Child/
14	teenager*.mp.
15	exp Pediatrics/ or p*diatric.mp.
16	12 or 13 or 14 or 15
17	11 and 16

### Eligibility criteria

Studies were eligible to be included if they provided details of the clinical or functional outcomes of a cohort of young people (from mid-late adolescence to early adulthood) who crossed the transition boundary of children’s mental health services, or if they provided details of the service pathway taken by a cohort of young people who crossed the transition boundary. Here, we define transition boundary as the upper age limit of a CAMHS. Conference abstracts were eligible to be included if the research had not been published elsewhere. There were no language restrictions in this review.

We did not include research involving the transition of young people with physical illnesses, neurological conditions (e.g., epilepsy), young people with a severe learning disability, or young people who were not transitioning in a mental health service. Case studies, editorials, literature or systematic reviews, opinion pieces, and policy documents were also excluded.

### Study selection

After de-duplication of references, titles and abstracts were screened by one reviewer (RA), and a random 10% were screened by another member of the research team (EF). Agreement was high between both reviewers (*k*_max_ = 0.85). Any references which met the inclusion criteria were then screened by full text by two reviewers independently (split between RA, EF, and CC). If the title and abstract did not contain sufficient information to decide on eligibility, then they were included for full-text screening. Any disagreement between reviewers was resolved through discussion.

### Quality assessment

Quality assessment of included studies was conducted independently by two reviewers (RA and CC) using a modified version of the Newcastle–Ottawa Scale [14]. All studies were included regardless of quality due to the lack of research in this area; however, the results of quality assessment were used to inform the narrative synthesis of results.

### Data extraction

A data extraction tool was piloted on a small number of included studies, modified, and then used to extract data from all studies. It included the following headings: year of publication, country of origin, aims, study design, sampling method, methodology, results, and how results were presented. Data extraction was carried out by two reviewers independently (RA and CC).

### Data synthesis

Data were synthesised narratively using steps adapted from Popay et al. [15]. These are: (1) to develop a preliminary synthesis of findings of included studies; (2) to explore relationships in the data; (3) to assess the robustness of the synthesis. A meta-analysis was not conducted due to the heterogeneity of the included studies.

## Results

### Study selection

**Fig. 1:**
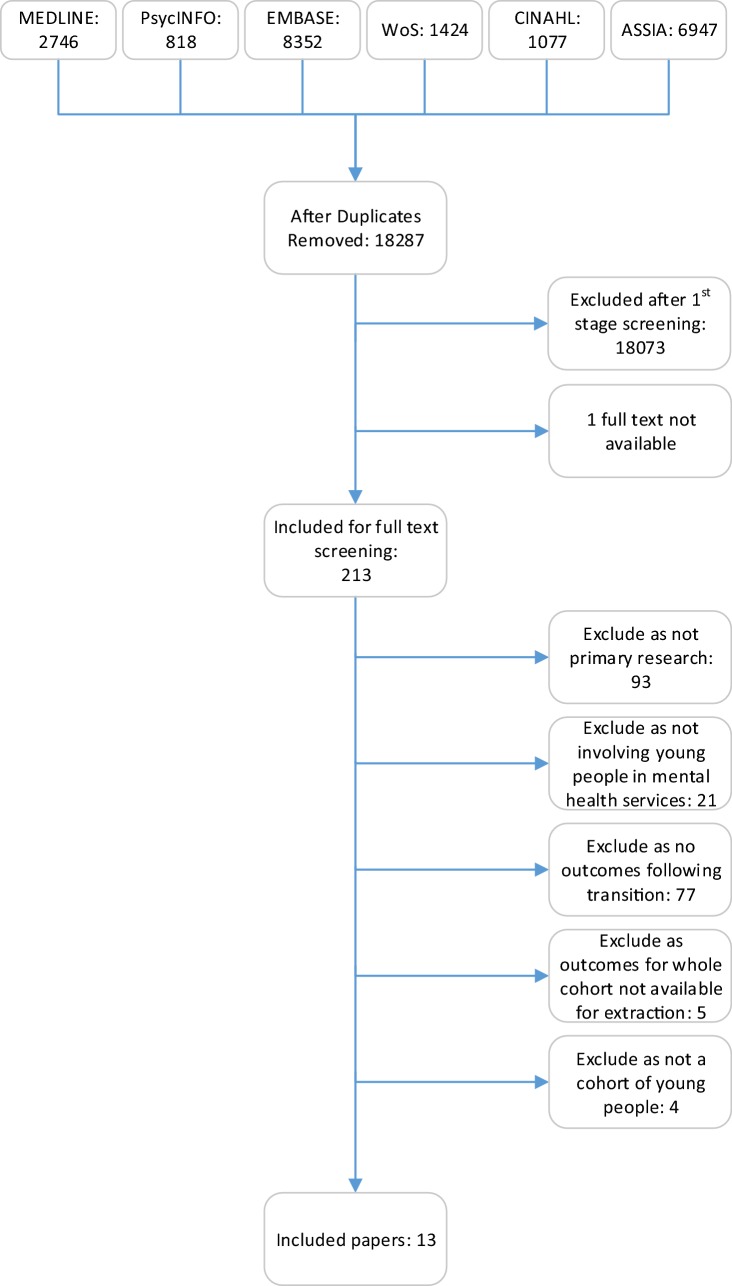
PRISMA flowchart showing screening of identified papers

After duplicates were removed, 18,287 studies remained for screening by title and abstract. 213 studies were included for full-text screening, of which 200 studies were excluded to leave 13 studies for inclusion in this review, representing 10 different cohorts of young people crossing the CAMHS transition boundary. Figure 1 illustrates the paper selection process. Only one study explored mental health outcomes after transition [16]; however, this data could not be extracted as CAMHS leavers were grouped with looked after children. Therefore, only information on service use outcomes following transition will be discussed in this review.

### Study characteristics

The 13 included studies represent research carried out in six different countries, Canada [17], England [2–4, 16, 18, 19], the Republic of Ireland [5, 20], France [21], Australia [22], and Italy [23, 24]. Two studies were service evaluations [17, 19]: one was a questionnaire study [24], one was a longitudinal study [16], and the remaining nine had a retrospective cohort study design [2–5, 18, 20–23]. Seven of the studies involved all young people in a cohort of CAMHS leavers, whilst four focused only on young people with attention-deficit/hyperactivity disorder (ADHD) [2, 19, 20, 24]. The sample sizes in the included studies ranged from 20 to 4226 young people. Table 2 shows further details of the included studies.

**Table 2:** Description of included studies

Study	Title	Country of origin	Study design	Age group	Participants	Results	Quality assessment
Cappelli et al. [17]	Transitioning youth into adult mental health and addiction services: An outcome evaluation of the youth transition project	Canada	Service evaluation	Young people (YP) between ages of 16 and 24	215 Young people (YP) accessing CAMHS who were referred to the transition programme	Young people (YP) who transitioned were more likely to have a higher score on a measure of antisocial behaviour (*x*^2^(1,59) = 3.84, *p* = 0.05) or have an anxiety disorder (*x*^2^(1,199) = 4.05, *p* = 0.044) than those who engaged with services. YP who transitioned as opposed to remaining on the waiting list had a higher number of visits to the emergency department (*x*^2^(1,115) = 4.76, *p* = 0.029) and reported more unmet needs relating to psychological distress (*x*^2^(3,106) = 10.98, *p* = 0.012). YP who remained on the waiting list were more likely to have oppositional defiant disorder (*x*^2^(1,200) = 7.64, *p* = 0.006) or ADHD (*x*^2^(1,200) = 4.83, *p* = 0.028)	Good
Islam et al. [18]	Mind how you cross the gap! Outcomes for young people who failed to make the transition from child to adult services	England	Retrospective case note analysis	YP who reached transition boundary during 12 month study period and had not been transitioned to AMHS after reaching the CAMHS age boundary	64 YP who had mental health needs, but were not transitioned to AMHS	The majority of YP who did not transition to AMHS were those with a diagnosis of an emotional or neurotic disorder (48.4%). The next most common diagnostic group who did not transition were those with a neurodevelopmental disorder (23.4%)	Good
McNicholas et al. [5]	Who is in the transition gap? Transition from CAMHS to AMHS in the Republic of Ireland	Republic of Ireland	Retrospective case note analysis	Cases included if YP were open to the service when they reached the upper age limit of that service in the 12 month study period	62 YP who crossed CAMHS transition boundary	Several of the YP with an ongoing mental health need (*n* = 47) were not referred to AMHS (45%). Refusal by the YP or their parent/carer was also a common reason for non-referral (23%). 32% of YP were referred to AMHS, with YP more likely to make the transition if they had a diagnosis of psychosis (*x*^2^(2,45) = 8.96, *p* = 0.02, *V* = 0.45). In contrast, YP with a diagnosis of ADHD were most likely to refuse the referral to AMHS (*x*^2^(2,45) = 6.81, *p* = 0.01, *V* = 0.44)	Good
Memarzia et al. [16]	Adolescents leaving mental health or social care services: predictors of mental health and psychosocial outcomes 1 year later	England	Longitudinal cohort study	YP aged 17–18 facing transition	26 YP who were about to leave CAMHS and 27 looked after children	Of the YP who left CAMHS, the majority (82%) were discharged to their GP, whilst 14% were referred to AMHS. Mental health outcomes were recorded, but could not be extracted for the CAMHS leavers, as data were grouped with the whole cohort	Good
Moosa and Sandhu [19]	Transition from children’s to adult services for patients with ADHD: a model of care	England	Service evaluation	Adolescents 15 or over with a diagnosis of ADHD who were open to CAMHS	247 YP with ADHD who were 15 or older in CAMHS	Before this scheme was introduced, 134 YP remained at CAMHS after they had reached the upper age limit of the service, which was reduced to 14 following its implementation. The referral rate to AMHS increased from 67 to 95% after the new initiative	Good
Ogundele [2]	Transitional care to adult ADHD services in a North West England District	England	Retrospective case note analysis	Adolescents with ADHD from childhood who were eligible for transition to AMHS, who reached 16 years old during study period	104 YP who were eligible for transition to AMHS	65% of YP were discharged from paediatric services without referral, often due to disengagement or self-discharge. 15% of YP were referred to an AMHS, and 18% to a CAMHS service. Of these YP referred to another service, 32% were discharged within the following 2 years	Poor
Paul et al. [3]	Transfers and transitions between child and adult mental health services	England	Retrospective case note analysis	YP who reached transition boundary during 12 month study period	154 YP who crossed transition boundary	Of the 131 YP with an ongoing clinical need, 102 were referred to AMHS and 90 were accepted. The most common reasons for non-referral were the CAMHS clinicians thinking AMHS would not accept the referral or not having an appropriate service to refer to, delayed referral, or refusal by the YP or their parent/carer	Good
Perera et al. [22]	Determinants of transition from child and adolescent to adult mental health services: A Western Australian Pilot Study	Australia	Retrospective case note analysis	CAMHS closed cases from 01/06/04 to 30/06/13 if YP were within transition age (16–21)	245 YP discharged from CAMHS at transition boundary	Four main transition pathways from CAMHS were identified: not referred, directly referred to AMHS, delayed referral to AMHS, and referred but not accepted. CAMHS diagnosis was associated with the likelihood of engagement at AMHS (*x*^2^(2) = 10.99, *p* < 0.001). In particular, YP with a neurotic disorder were less likely to be engaged at AMHS than those with a mood or other types of disorder (all *z*s ≥ 2.25, *p*s ≤ 0.01)	Good
Reale et al. [24]	Transition to adult mental health services for young people with attention-deficit/hyperactivity disorder in Italy: parent’s and clinician’s experiences	Italy	Qualitative questionnaire study	Parents of adolescents with ADHD who reached adulthood—identified through mailing list of support group	Parents and clinicians of YP with ADHD representing 24 young adults	Results showed that the most common outcome following transition from the children’s service was no ongoing care (38%), excluding the YP who had been discharged because of good health (12%). No YP had been referred to the adult service from their children’s service, although 21% were receiving care in a public adult mental health service and 17% by a private specialist. A further 12% of YP continued to receive care at the children’s service after crossing the transition boundary	Poor
Schandrin et al. [21]	Transition from child to adult mental health services: a French retrospective study	France	Retrospective case note analysis	Every patient whose transition from CAMHS to AMHS was initiated at a hospital during the 2 year study period	31 YP who transition had been initiated	Transition was completed in 90% of cases; however, YP often experienced discontinuity of care during their transition with an average gap of 3 months of no care between the services. At 3 months following transition to AMHS, 84% were actively engaged, although this fell to 45% at one to 3 years later	Poor
Singh et al. [4]	Process, outcome and experience of transition from child to adult mental healthcare: multiperspective study	England	Retrospective case note analysis	See Paul et al. [3]	154 YP who crossed transition boundary	YP were more likely to be referred to AMHS if they had been admitted under the Mental Health Act (OR 5.0; 95% CI clustered 1.6–15.5; *p* = 0.01), had a severe and enduring mental illness (OR 2.82; 95% CI clustered 0.8–9.6; *p* = 0.01), were on medication at the time of transition (OR 7.85; 95% CI clustered 1.5–40.9; *p* = 0.01), or had a comorbidity (OR 2.36; 95% CI clustered 1.7–3.4; *p* < 0.01). YP with emerging personality disorder were reported to be more likely to fall through the gap; however, the numbers were too small to draw statistically significant conclusions	Good
Stagi et al. [23]	Continuity of care from child and adolescent to adult mental health services: evidence from a regional survey in Northern Italy	Italy	Retrospective case note analysis	YP aged 16 or older listed in a health database as having attended CAMHS in a 3 year period. Received formal diagnosis	8239 YP who crossed the CAMHS transition boundary	Over the 4 year study period, 19.4% of YP were transferred to AMHS. YP were more likely to make this transition if they had a diagnosis of schizophrenia or related disorders (OR 3.92; 95% CI 2.17–7.08), a personality disorder (OR 2.69; 95% CI 1.89–3.83) or a pervasive developmental disorder (OR 2.13; 95% CI 1.51–2.99). Of the 2771 YP referred to AMHS, 580 were accepted and an additional 241 received joint care from both services	Good
Tatlow-Golden et al. [20]	Transitioning from child and adolescent mental health services with attention-deficit/hyperactivity disorder in Ireland: case note review	Republic of Ireland	Retrospective case note analysis	See McNicholas et al. [5]. Sample of YP with ADHD included in this paper	20 YP who crossed CAMHS transition boundary and had a diagnosis of ADHD	None of these YP were directly referred to a public AMHS	Good

### Risk of bias

The quality of the included studies varied, with 10 being of good quality and three being of poor quality (see Table 2 for more details). Studies were rated as poor if they did not include a measure of clinical need to transition or a breakdown of transition for different subgroups (e.g., different diagnoses, age groups, severity of illness, etc.) and if detailed baseline information of the cohort was missing.

### Synthesis of results

The synthesis of individual study findings shows a care gap at the end of CAMHS, with only 24% of young people transitioning to AMHS after reaching their CAMHS age boundary (see Table 3 for details). Three studies [4, 5, 16] explored the service use destinations of young people who had an ongoing clinical need at the end of CAMHS and found that some did not receive an AMHS referral, despite still being judged to need ongoing care, with figures ranging from 42 to 84% (the latter figure includes some looked after children in Memarzia et al. [16]). In addition, four studies [19, 20, 22, 24] showed that 103 young people were discharged from CAMHS, only for them to be referred to AMHS by their GP.

**Table 3:** Service use outcomes of young people crossing the CAMHS transition boundary (number of young people)

Service use pathway after crossing transition boundary	Cappelli et al. [17]	TRACK cohort Islam et al. [18], Paul et al. [3], Singh et al. [4]	ITRACK cohort McNicholas et al. [5]	ITRACK cohort—long-term ADHD outcomes Tatlow-Golden et al. [20]	Memarzia et al. [16]	Moosa and Sandhu [19]	Ogundele [2]	Perera et al. [22]	Reale et al. [24]	Schandrin et al. [21]	Stagi et al. [23]	Total
Transition to AMHS	127	90	14	-	3	94	16	62	-	28	821	1255
Engaged at AMHS^a^	-	76	-	-	-	-	13	-	-	26	-	115
Not referred—discharged	-	3	9	-	-	-	-	-	-	-	-	12
Not referred—unknown	-	4	-	1	4	-	-	88	-	-	-	93
Not referred—CAMHS	-	-	12	1	-	80	1	-	3	-	1214	1311
Not referred—other^b^	-	20	-	-	1	9	-	-	-	-	-	30
Refused—disengaged	-	-	2	3	-	-	-	-	-	-	-	5
Refused—discharged	-	11	3	4	-	-	-	-	-	-	-	18
Refused—CAMHS	-	-	6	-	-	-	-	-	-	-	-	6
Unsuccessful AMHS transition	-	-	1	-	-	-	-	29	-	-	2191	2221
Unsuccessful—discharged	-	2	-	-	-	-	-	-	-	-	-	2
Unsuccessful—CAMHS	-	2	-	-	-	-	-	-	-	1	-	3
Unsuccessful—CAMHS–GP	-	3	-	-	-	-	-	-	-	-	-	3
Discharged to GP (well)	-	9	15	3	-	-	-	-	3	-	-	30
Discharged to GP	-	-	-	-	18	-	68	-	9	-	-	95
Discharged then AMHS	-	-	-	1	-	31	-	66	5	-	-	103
Disengaged	47	5	-	5	-	33	-	-	-	2	-	92
Private	-	-	-	2	-	-	-	-	4	-	-	6
Other CAMHS	-	-	-	-	-	-	19	-	-	-	-	19
Transition pending	41	5	-	-	-	-	-	-	-	-	-	46
Total	215	154	62	20	26	247	104	245	24	31	4226	5350

Numbers will not add up to column total

^a^Young people attended at least 1 appointment

^b^Other reasons recorded include: uncertain asylum status, care ending, and multiple reasons

A quarter of young people remained at CAMHS after crossing the transition boundary, whilst another quarter transitioned to AMHS. The other 50% had varied service use destinations; however, in most studies, the follow-up periods were not long enough to find out what happened to these young people after being discharged from CAMHS. Disengagement was high, with all but four studies [16, 22–24] including disengagement as an outcome after young people left care at CAMHS. The number of young people who were discharged due to disengagement was recorded in all but one study [2], with disengagement ranging from 3 to 40% of young people.

Two studies [3, 5] reported young people not being referred to AMHS, because CAMHS clinicians did not think that young people would meet the inclusion criteria or that AMHS did not have the necessary expertise. Five studies recorded unsuccessful referrals to AMHS [5, 18, 21–23], with percentages of referrals rejected ranging from 3 to 73%. Full details of young people’s service use outcomes following reaching the upper age limit of their CAMHS service is shown in Table 3.

### Optimal transition

Three studies evaluated how many young people experienced optimal transition, two [4, 21] according to the four principles of ‘optimal transition’ identified by Paul et al. [3]. In most cases, optimal transition was not achieved, with percentages of young people having optimal transition recorded at 6% [16], 13% [21], and 4% [4].

### Waiting times

Three studies explored the average waiting times which young people experienced during their transition to AMHS [5, 17, 21]. All found that young people experienced long delays, ranging from 55 to 110 days.

### AMHS engagement

Three studies looked at engagement at AMHS following transition, the TRACK study (as reported by [3, 4]), Ogundele [2], and Schandrin et al. [21]. Of the 134 young people in these studies who transitioned to AMHS, 115 (86%) had at least one appointment. Rates of engagement fell further after this first appointment, with 16% being discharged after one AMHS appointment in the TRACK study [4] and 55% being discharged in the 1–3 years following transition in the study by Schandrin et al. [21].

### Outcomes of young people with ADHD

Four studies focused on young people with ADHD. One was a service evaluation following improvements to their transition process [19], and this showed a much higher rate of transition to AMHS (38%) than the other three studies carried out in the standard care (11%). In two of the studies involving young people with ADHD, none of the cohort was transitioned to an AMHS at the CAMHS age boundary [20, 24]. Of the young people who were discharged to their GP following cessation of care in CAMHS, one-third were then referred to an AMHS, implying that they were discharged despite having an ongoing clinical need for treatment.

## Discussion

The aim of this review was to synthesise the existing research on mental health and service use outcomes of young people after leaving CAMHS. Thirteen studies were included, all of which reported service use destinations of young people after leaving CAMHS. Only one study included mental health outcomes after transition; however, as these data were reported for the whole cohort which included young people leaving care, this review focuses on service use outcomes after leaving CAMHS.

The included studies show the wide range of service use destinations of young people who reach the upper age limit of CAMHS, with only around a quarter of young people continuing care in AMHS. Alternative destinations included: other CAMHS services, community-based services, private care, or transfer of care to a GP. There are a variety of different pathways taken by young people, and multiple changes of service are common during this transition period. A quarter of young people stayed in CAMHS despite reaching the upper age limit of that service, either due to non-referral or their referral to AMHS not being accepted. This high variability in transition outcomes reflects the different ways which CAMHS services are funded and organised in different countries, as well as the availability of appropriate AMHS [1, 26]. In addition to variation between countries, there was also significant variation in outcomes between participants studied at a national level, in the United Kingdom. These results indicate that young people receive differing quality of care depending on where they live, with different service models and transition boundaries.

There was also evidence to show that some young people experienced high disruption during the transition period: some were not referred onwards despite still requiring treatment when they crossed the CAMHS age boundary, whilst very few of those who did transition received optimal transitional care. This suggests that young people were poorly prepared for transition and experienced poor continuity of care, something echoed in several research studies exploring young people’s experiences of transition (e.g., [7, 25]). Having a poor transition experience could result in poor engagement with the adult service [11], which is supported by the findings in this review as studies showed the high levels of disengagement. Young people may also find it difficult to engage with AMHS due to the significant difference in focus and culture between the two services, something which has been identified as a potential barrier to young people’s engagement in continued mental health care [10]. The results of this review suggest that services are not following the current guidance for the best practice, which states that transition planning should be started early and in conjunction with the young person, whilst taking into account their need for ongoing support and at what point transition would be most appropriate [13]. Moving forward, services should aim to align clinical practice with the current mental health policy to provide the best possible care for young people as they reach the upper age limit of CAMHS.

Four of the included studies focused on young people with ADHD, as young people with this diagnosis are among the groups least likely to transition to AMHS [4]. In two of these studies, none of the young people were transitioned directly to AMHS, although a minority were referred to adult services by their GP or received private care after leaving CAMHS [20, 24]. This could reflect a lack of appropriate service provision in some areas, leaving CAMHS with no choice but to discharge the young person to their GP [27]. In contrast, the service improvement study by Moosa and Sandhu [19] reported much higher rates of transition, suggesting that AMHS will accept the referrals of young people with ADHD, providing that the transition is managed effectively.

As several young people were not transitioned directly to AMHS, but, instead, first discharged to a GP, it can be argued that they did not receive sufficient continuity of care during their transition between services. Studies did not explore why a direct transfer of care was not made. A further clinical implication of this review is the finding that around a quarter of young people studied remained at CAMHS, even after reaching the upper age limit for that service. In this case, CAMHS should receive the appropriate funding and resources to provide this ongoing care, without restricting their ability to accept new referrals. One way in which mental health services have responded to the need for streamlined care has been to introduce new 14–25 services, removing the traditional transition boundary at around 16–18 years of age [28]. The initial findings have indicated that this new service model can help to reduce the number of young people experiencing an abrupt end to their care when they reach 18 [29]. However, in order for these services to operate effectively, appropriate funding and resources are needed to ensure that other service users do not suffer as a result.

## Implications for future research

This review has also highlighted gaps in the existing research regarding service use outcomes of young people who reach the upper age limit of CAMHS, in particular longitudinal research which includes longer term outcomes in the months or years after transition. In recent years, new transition guidelines have been released; however, we are unable to fully assess what impact these guidelines have had on clinical practice due to the lack of research in this area. More longitudinal research is required to fully understand how these guidelines have been incorporated into practice and what impact they have had on the transition experiences of young people. The mental health outcomes of young people following transition are also currently unknown, something which should be made a priority in future research.

## Strengths and limitations

To our knowledge, this is the first review which has systematically synthesised evidence for the service use destinations of young people after they have reached the upper age limit of CAMHS. This review has systematically collated and critically evaluated transition research from six different countries, giving a picture of transition outcomes across high-income countries. A particular strength of the methodology employed was the use of wide search criteria to minimise chances of missing relevant research. Searches also included grey literature and had no language restrictions. However, not all of the studies included were of a high methodological quality, and therefore, there are some limitations which should be considered during the interpretation of these results.

First, poor record keeping by the mental health services in some of the studies meant that the service use outcomes of some cases were unknown. Poor record keeping in some services also led to differences in the selection method of cases; some used record linkage, whilst others used clinicians to retrospectively identify eligible cases as records were not available. It is possible that cases with a particularly good or bad transition were more likely to be remembered which could lead to bias in the sample. A further limitation is that some studies did not report long-term outcomes; for example, they did not show what happened to young people whose transition was recorded as ‘pending’, those who stayed in CAMHS, or those whose referral to AMHS was unsuccessful.

Details about a young person’s mental health and illness severity were also missing from some studies. For example, not all studies evaluated ongoing clinical need at the transition boundary; in some cases, this was not mentioned, whilst, in others, having a diagnosis of mental illness was enough to imply an ongoing need. Therefore, we cannot draw firm conclusions regarding the true numbers of young people who were not transitioned to AMHS despite still being unwell and needing further care. Similarly, not all studies distinguished between young people who were discharged to their GP, because they were well and so no longer needed treatment, those discharged to GP for continued medical review, and those who were discharged to their GP, because there was no appropriate service for them to transition to.

Finally, heterogeneity between the analyses in the different studies meant that quantitative synthesis of results using a meta-analysis was not appropriate.

## Conclusions

Systematic review of the literature revealed that only a quarter of young people continued to access care at AMHS after reaching the upper age limit of CAMHS. The remainders have varied service use outcomes, characterised by multiple transitions during this period. Future research should record the long-term outcomes of CAMHS leavers, both in terms of whether they continue to receive care and their mental health and functioning outcomes after transition.

## Appendix

A breakdown of quality assessment scores for each paper Table 4. The number of stars etc.

**Table 4:** The number of stars scored in each domain of the Newcastle–Ottawa scale for each included paper

Study	Selection (*/4)	Comparability (*/2)	Outcome (*/3)
Cappelli et al. [17]	3*	2*	2*
Islam et al. [18]	3*	2*	3*
McNicholas et al. [5]	3*	2*	3*
Memarzia et al. [16]	4*	1*	2*
Moosa and Sandhu [19]	3*	2*	3*
Ogundele [2]	3*	0*	3*
Paul et al. [3]	3*	2*	3*
Perera et al. [22]	4*	1*	3*
Reale et al. [24]	2*	0*	1*
Schandrin et al. [21]	4*	0*	2*
Singh et al. [4]	3*	2*	3*
Stagi et al. [23]	3*	1*	2*
Tatlow-Golden et al. [20]	3*	2*	3*
